# Dopaminergic Projections From the Ventral Tegmental Area to the Nucleus Accumbens Modulate Sevoflurane Anesthesia in Mice

**DOI:** 10.3389/fncel.2021.671473

**Published:** 2021-04-30

**Authors:** Huan Gui, Chengxi Liu, Haifeng He, Jie Zhang, Hong Chen, Yi Zhang

**Affiliations:** ^1^Department of Anesthesiology, The Second Affiliated Hospital of Zunyi Medical University, Zunyi, China; ^2^Guizhou Key Laboratory of Anesthesia and Organ Protection, Affiliated Hospital of Zunyi Medical University, Zunyi, China; ^3^School of Anesthesiology, Zunyi Medical University, Zunyi, China

**Keywords:** ventral tegmental area, dopaminergic (DA) neuron, calcium fiber photometry recording, dopamine sensors, sevoflurane

## Abstract

The role of the dopaminergic pathway in general anesthesia and its potential mechanisms are still unknown. In this study, we usedc-Fos staining combined with calcium fiber photometry recording to explore the activity of ventral tegmental area (VTA) dopaminergic neurons(VTA-DA) and nucleus accumbens (NAc) neurons during sevoflurane anesthesia. A genetically encoded dopamine (DA) sensor was used to investigate thefunction of the NAc in sevoflurane anesthesia. Chemogenetics and optogenetics were used to explore the role of the VTA-DA in sevofluraneanesthesia. Electroencephalogram (EEG) spectra, time of loss of righting reflex (LORR) and recovery of righting reflex (RORR) were recorded asassessment indicators. We found that VTA-DA and NAc neurons were inhibited during the induction period and were activated during the recoveryperiod of sevoflurane anesthesia. The fluorescence signals of dopamine decreased in the induction of and increased in the emergence from sevoflurane anesthesia.Activation of VTA-DA and the VTA^DA^-NAc pathway delayed the induction and facilitated the emergence accompanying with thereduction of delta band and the augmentation of the gamma band. These data demonstrate that VTA-DA neurons play a critical role in modulating sevofluraneanesthesia *via* the VTA^DA^-NAc pathway.

## Introduction

General anesthesia is characterized by unconsciousness, analgesia, amnesia, and immobility while maintaining vital physiological functions, but how anesthetic drugs create a state of unconsciousness remains unclear. Growing evidence has demonstrated that general anesthesia and sleep-wakefulness share homologous neural substrates (Nelson et al., 2002; Franks, 2008; Franks and Zecharia, 2011). Dopaminergic nuclei have been confirmed to be involved in the processes of both sleep-wake (Oishi and Lazarus, 2017; Qiu et al., 2019) and general anesthesia (Harrington, 2014; Li et al., 2019), in which the ventral tegmental area (VTA) dopaminergic (DA) neurons are specifically crucial. It has been suggested that the VTA promotes rapid eye movement (REM) sleep and waking from natural sleep (Leung et al., 2014; Taylor et al., 2016; van der Meij et al., 2019). Lesions of VTA dopaminergic neurons result in a significantly prolonged recovery time from propofol (Zhou et al., 2015). Furthermore, electrical stimulation or optogenetic activation of VTA-DA neurons induce fast awakening from isoflurane anesthesia (Solt et al., 2014; Taylor et al., 2016). These studies indicate that the VTA plays an important role in sleep regulation and general anesthesia. However, it remains to be determined which VTA downstream pathways are involved in the mechanism of general anesthesia.

Projections from VTA-DA neurons to the nucleus accumbens (NAc), which is known as the main mesolimbic dopaminergic pathway, play a key role in depression (Nestler and Carlezon, 2006), addiction (Shen et al., 2016), feeding behavior (Skibicka et al., 2013) and sleep-wake circuits (Eban-Rothschild et al., 2016; Oishi et al., 2017). NAc dopaminergic receptors are likely involved in the maintenance of wakefulness (Luo et al., 2018). Using intracerebral microdialysis, Léna et al. (2005) show that extracellular dopamine levels in the NAc are higher during arousal states than during slow-wave sleep. In addition, our previous studies indicate that microinjection of a D1 receptors (D1R) agonist into the NAc shell accelerates the emergence process from isoflurane anesthesia in mice (Zhang et al., 2020). Now, the most commonly used volatile anesthetic agent is sevoflurane which is an ideal drug for its pleasant smell, non-irritant effect on the airways and low blood-gas partition coefficient, especially for children (Boonmak et al., 2016; Brioni et al., 2017; Xu et al., 2020) Therefore, we speculate that VTA-DA neurons modulate the process of sevoflurane-induced general anesthesia through projections to the NAc. To test this hypothesis, we applied c-Fos staining, calcium fiber photometry recordings, optogenetics, chemogenetics, and fluorescent sensors, combined with behavioral tests and electroencephalography (EEG)analysis, to investigate the roles of the VTA^DA^-NAc pathway in the induction of and emergence from sevoflurane anesthesia.

## Materials and Methods

### Animals

This study was performed in accordance with the guidelines described in the Guide for the Care and Use of Laboratory Animals in China (No. 14924, 2001) and was approved by the Animal Care and Use Committees of Zunyi Medical University. Adult male DAT-cre mice (No. 006660; Jackson Laboratory, Bar Harbor, ME, USA) and C57BL/6 mice (SCXK2019-0014; Tianqin, Changsha, China) were used in this study. Before all experiments were performed, all mice were in good health and at a normal weight (22 g to 28 g). Mice were housed in standard chambers within an SPF laboratory animal room (12/12-h light/dark cycle; 23 ± 2°C; relative humidity: 55% ± 2%). Adult (8–12 weeks) mice were used for all the experiments.

### Drugs

Sevoflurane and isoflurane were purchased from RWD Life Science (Shenzhen, China). The rabbit anti-tyrosine hydroxylase antibody (Ab-112, USA) was purchased from Abcam Corporation (Cambridge, UK). The rabbit c-Fos antibody (2250S, USA) was purchased from Cell Signaling (Beverly, MA, USA). The secondary antibodies were goat anti-rabbit conjugated to Alexa 488 (Invitrogen; A-11008) and goat anti-rabbit conjugated to Alexa 594 (Invitrogen; A-11012, Carlsbad, CA, USA).

### Stereotaxic Surgery

Isoflurane (1.4%) and oxygen (1 L/min) were applied to anesthetize mice in a small box. Before surgery, lidocaine (2%) was injected subcutaneously to induce local anesthesia. Subsequently, the mice were gently placed on a stereotaxic frame (RWD Life Science, Shenzhen, China). Hydrogen peroxide (3%) was used to remove the fascia from the skull surface. The bregma and lambda points were used to adjust the mouse head to the horizontal position. A small window with a diameter of 300–500 μm was opened above the point of virus injection and fiber implantation. All viruses purchased from Brain-VTA (Wuhan, China). An adeno associated virus expressing GCaMP (rAAV2/9-hSyn-DIO-GCaMP6s, PT-0091; rAAV2/9-hSyn-GCaMP6s, PT-0145), DA2h (rAAV9-hSyn-DA2h, PT1301), chemogenetic (rAAV2/9-EF1α-DIO-hM3Dq-EGFP, PT-0042; rAAV2/9-EF1α-DIO-hM4Di-EGFP, PT-0043), optogenetic (rAAV2/9-EF1α-DIO-ChR2-mCherry, PT-0002; rAAV2/9-EF1α-DIO-NpHR-mCherry, PT-0007), Retro-cre (rAAV2/Retro-hSyn-cre-mCherry, PT-0407), mCherry (rAAV-Ef1α-DIO-mCherry-WPRE-pA, PT-0013), and EGFP (rAAV-Ef1α-DIO-EGFP-WPRE-pA, PT-0012) was injected into the VTA (anterior-posterior [AP]: −3.10 mm, medial-lateral [ML]: +0.8 mm, and dorsal-ventral [DV]: −4.3 mm) or NAc (AP: +1.45 mm, ML: +0.7 mm, DV: −4.45 mm) *via* a glass micropipette using a microsyringe pump. The injection was guided by the stereotactic coordinates of the mouse brain (George Paxinos and Keith B. J. Franklin, second edition). A total of 200–250 nl of virus was delivered to each site over 10 min *via* a microsyringe pump, after which the needle was left in the injection position for at least 10 min to permit diffusion. For calcium fiber photometry, DA dynamics monitoring and optogenetic testing, optic fibers were implanted unilaterally over the NAc and secured with three skull screws and dental cement. All animals were individually housed and allowed to recover completely before the formal experiment.

### Calcium Fiber Photometry Recording

**Figure 1:**
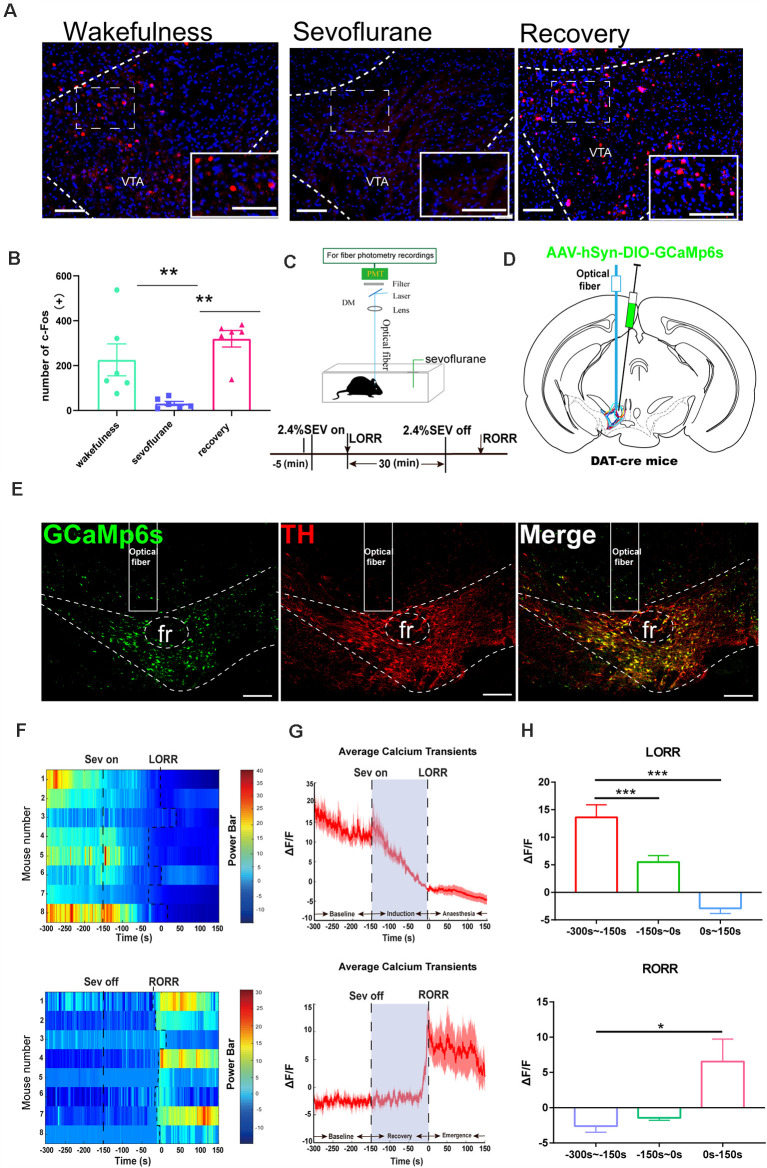
Ventral tegmental area (VTA)-dopamine (DA) activity and neural dynamics in response to sevoflurane. **(A)** Expression of c-Fos in the VTA in the wakefulness state, sevoflurane anesthesia and recovery from anesthesia in mice (Scale bars: 200 μm). **(B)** Average number of c-Fos-immunopositive neurons. One-way analysis of variance (ANOVA) followed by *post hoc* Bonferroni’s test (*n* = 6). **p < 0.01. **(C)** Schematic configuration of *in vivo* Ca^2+^ signal recording. Timeline for Ca^2+^ signal recording in sevoflurane anesthesia. **(D)** Expression of GCaMP6s in VTA from each cases are outlined. **(E)** Expression of GCaMP6s in VTA-DA neurons. Viral expression (GCaMP6s, green) in the VTA and colabeling with DA neurons (TH immunofluorescence, red; Scale bars: 200 μm). **(F)** Heat map illustrating changes in the Ca^2+^ signals related to sevoflurane-induced LORR and RORR. **(G)** Average calcium transients associated with loss of righting reflex (LORR) and recovery of righting reflex (RORR) mean (red trace) ± SEM (red shading). Note that the Ca^2+^signals gradually decreased during the LORR process and increased sharply after RORR. **(H)** ΔF/F represents the deviation in Ca^2+^ fluorescence from the baseline, which is the averaged ΔF/F between *t* = –300 s and –150 s. Data are presented as the mean ± SEM. *p < 0.05; **p < 0.01; ***p < 0.001, *n* = 8, by paired *t*-test.

As a calcium indicator, GCaMP6s can reflect transient alterations of neuronal activity with high sensitivity. In the current experiment, *in vivo* Ca^2+^ signals of the VTA and the NAc were recorded under sevoflurane anesthesia (2.4%) mixed with oxygen (1 L/min) using a multichannel fiber photometry system (Thinker-Tech Nanjing Bioscience Nanjing, China), which was equipped with a 480 nm excitation LED (3 W, CREE) and a dichroic mirror (DCC3420M; Thorlabs). An optical fiber (Newton Inc., China) integrated with an optical diverter (Doric Lenses) was used to transmit light between the fiber photometry system and the implanted optical fiber (Figures 1C, 4C).

**Figure 2:**
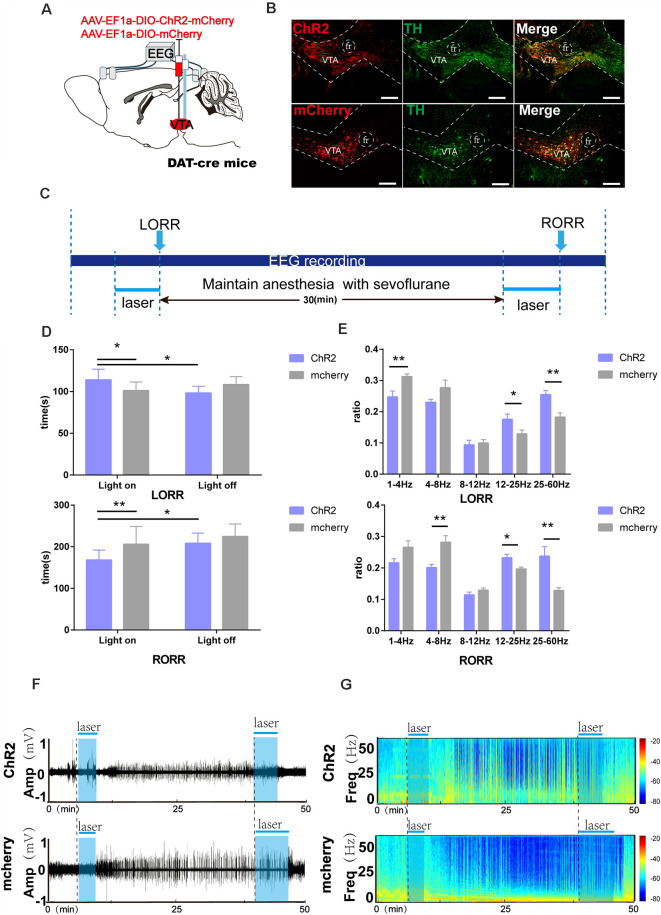
Optogenetic activation of VTA-DA neurons facilitates arousal from sevoflurane anesthesia. **(A)** Diagram of the optogenetic virus injection and stimulation sites. **(B)** Expression of virus (mCherry, red) in VTA-DA neurons and colabeling with DA (TH immunofluorescence, green; scale bar: 200 μm). **(C)** Schematic of the experimental time-course. **(D)** Optical activation of DA neurons in the VTA prolonged the induction time and shortened the emergence time from sevoflurane anesthesia. **(E)** Comparison of each Electroencephalogram (EEG) frequency band between the two groups during optogenetic activation of VTA-DA neurons. **(F,G)** Representative EEG traces and the corresponding heat map of the process in the two groups. Data are presented as the mean ± SD. *p < 0.05; **p < 0.01, *n* = 8, by paired and unpaired *t*-test.

**Figure 3:**
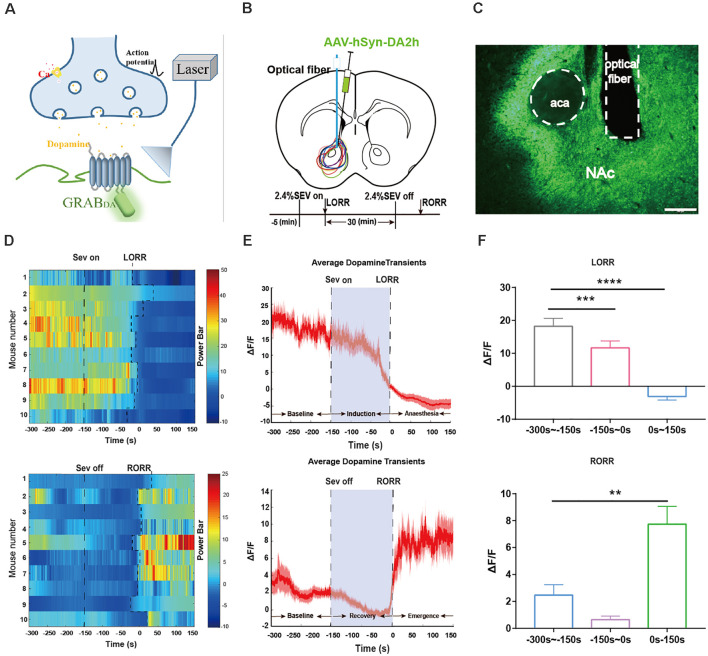
Dynamics of extracellular dopamine in the nucleus accumbens (NAc) in response to sevoflurane anesthesia. **(A)** Schematic diagram depicting the fiber photometry recording of extracellular dopamine. **(B)** Expression of DA2 h in NAc from each cases are outlined. Timeline for dynamics of extracellular dopamine recording in sevoflurane anesthesia. **(C)** Expression of the DA2h virus in the NAc area (Scale bars: 200 μm). **(D)** Heat map illustrating the changes in the DA fluorescence signals related to sevoflurane-induced LORR and RORR. **(E)** Average DA transmitters transients associated with LORR and RORR; mean (red trace) ± SEM (red shading). Note that the extracellular DA fluorescence signals gradually decreased with LORR and increased sharply after RORR. **(F)** ΔF/F represents the deviation in the DA transmitter signal from the baseline, which is the averaged ΔF/F between *t* = –300 s and –150 s. Data are presented as the mean ± SEM. **p < 0.01; ***p < 0.001; ****p < 0.0001, *n* = 10, by paired *t*-test.

**Figure 4:**
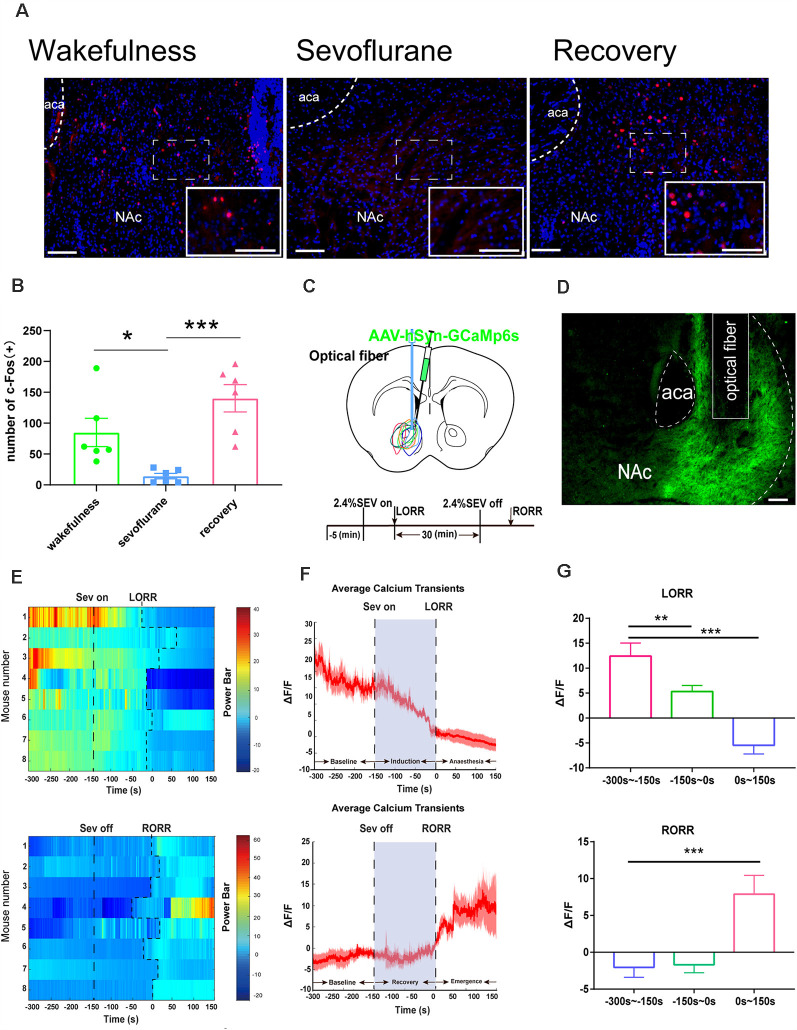
Nucleus accumbens (NAc) neuron activity and neural dynamics in response to sevoflurane. **(A)** Expression of c-Fos in the NAc in the wakefulness state, sevoflurane anesthesia and recovery from anesthesia in mice (Scale bars: 200 μm. **(B)** Average number of c-Fos-immunopositive neurons. One-way ANOVA followed by *post hoc* Bonferroni’s test. *n* = 6, *p < 0.05, ***p < 0.0001. **(C)** Expression of GCaMP6s in NAc neurons. **(D)** Expression of the DA2h virus in the NAc area (Scale bars: 200 μm). **(E)** Heat map illustrating the changes in the Ca^2+^ signals related to sevoflurane-induced LORR and RORR. **(F)** Average calcium transients associated with LORR and RORR; mean (red trace) ± SEM (red shading). **(G)** ΔF/F represents the deviation in Ca^2+^ fluorescence from the baseline, which is the averaged ΔF/F between *t* = –300 s and –150 s. Data are presented as the mean ± SEM. *p < 0.05, **p < 0.01, *n* = 8, by paired *t*-test.

The loss of righting reflex (LORR) and recovery of righting reflex (RORR) times are widely considered to be standard indices for assessing the processes of induction of and emergence from general anesthesia in mice, respectively. Animals were housed under free conditions for 10 min, and then, a 300 s recording was taken as a baseline. Next, the mice were anesthetized with sevoflurane (2.4%) with oxygen (1 L/min), and sevoflurane anesthesia was maintained for 30 min before discontinuation. The recording was stopped 5 min after RORR. All brains were sectioned to verify viral fluorescence expression and the position of optical fiber implantation after the experiments were completed. Fiber photometry data were analyzed using MATLAB 2019a (MathWorks, Cambridge, UK). The values of the fluorescence change (ΔF/F) were calculated using the following formula: (F − F0)/F0, where F is the test fluorescence signal and F0 is the average fluorescence intensity before stimulation (Liu et al., 2021).

### Monitoring DA Transmitters Release

We applied genetically encoding GPCR activation-based DA (GRABDA) sensors that respond to nanomolar and micromolar concentrations of dopamine. GRABDA sensors reflect the dynamic accumbens DA transmitters concentration in the NAc by coupling conformationally sensitive circular-permutated EGFP (cpEGFP) to specific DA receptors (Sun et al., 2018; Figure 3A). DA2h were expressed by adeno-associated virus (AAV) into NAc of C57BL/6 mice (Figures 3B,C). Alterations of the DA transmitters amount were reflected by the fluorescence intensity with high sensitivity, which was detected by a multichannel fiber photometry system. The protocol for recording the NAc extracellular DA transmitters’ concentration was identical to that for the calcium fiber photometry recording experiment.

### Chemogenetic Activation/Inhibition of VTA-NAc Dopaminergic Projections

**Figure 5:**
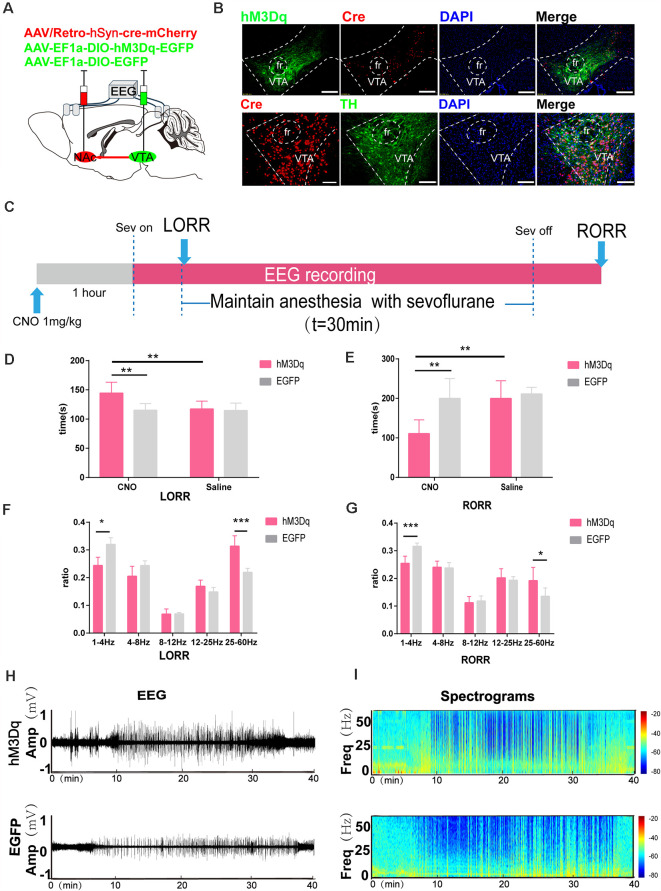
Chemogenetic activation of VTA-NAc pathway neurons facilitate emergence from sevoflurane anesthesia. **(A)** Schematic of the experimental protocol. Cre-dependent hM3Dq was injected into the VTA of mice, while retrograde cre was injected into the NAc. **(B)** Expression of hM3Dq-EGFP (green) and retrograde cre-mCherry (red) in the VTA and NAc showing dopaminergic projections from the VTA to the NAc in the mouse brain (above). Scale bars: 200 μm. Immunofluorescence of cre-mCherry (red) and TH (green) in the VTA (below) Scale bars: 100 μm. **(C)** Experimental timeline of the behavioral test and EEG recording. **(D)** CNO-mediated hM3Dq activation notably prolonged the LORR time. **(E)** CNO-mediated activation significantly shortened the RORR time. **(F)** CNO-mediated hM3Dq activation decreased δ bands and enhanced γ bands during the LORR process. **(G)** CNO-mediated hM3Dq activation decreased δ bands and enhanced γ bands during the RORR process. **(H,I)** Representative EEG traces and heat map from the two groups. CNO: clozapine N-oxide. Data are presented as the mean ± SD. *p < 0.05, **p < 0.01, and ***p < 0.001, *n* = 8, by paired and unpaired paired *t*-test.

**Figure 6:**
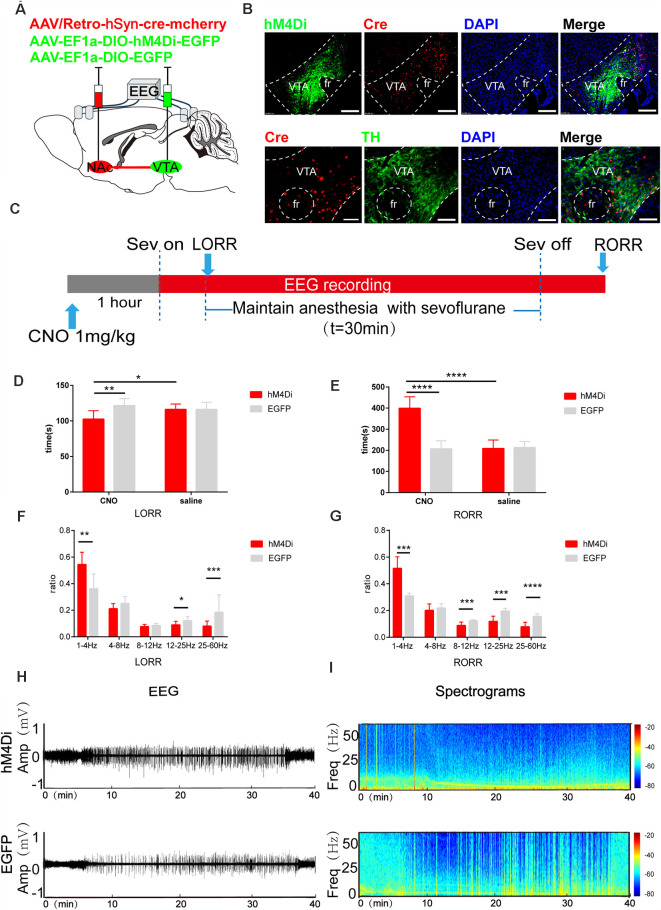
Chemogenetic inhibition of the VTA-NAc pathway regulates the induction and emergence processes of sevoflurane anesthesia. **(A)** Schematic of the experimental protocol. Cre-dependent hM4Di was injected into the VTA of mice, while retrograde cre was injected into the NAc. **(B)** Expression of hM4Di-EGFP (green) and retrograde cre-mCherry (red) in the VTA and NAc shows dopaminergic projections from the VTA to the NAc in the mouse brain (above). Scale bars: 200 μm. Immunofluorescence of cre-mCherry (red) and TH (green) in the VTA (below). Scale bars: 100 μm. **(C)** Experimental timeline of the behavioral test and EEG recording. **(D)** CNO-mediated hM4Di inhibition notably reduced the LORR time. **(E)** CNO-mediated hM4Di inhibition significantly prolonged the RORR time. **(F)** CNO-mediated inhibition increased the ratio of the δ band but reduced the ratio of the β and γ bands during LORR. **(G)** CNO-mediated inhibition led to an increased power of the δ band and decreased powers of the α, β and γ bands during RORR. **(H,I)** Representative EEG traces and heat map from the two groups. CNO: clozapine N-oxide. Data are presented as the mean ± SD. *p < 0.05, **p < 0.01, ***p < 0.001 and ****p<0.0001, *n* = 8, by paired and unpaired *t*-test.

To identify the anatomical and functional interaction between VTA-NAc dopaminergic projections during sevoflurane anesthesia, we separately performed rAAV/Retro-hSyn-cre-mCherry-WPRE-hGH retrograde tracing in the NAc and chemogenetic (rAAV-EF1α-DIO-hM3Dq-EGFPand rAAV-EF1α-DIO- hM4Di-EGFP) analysis in the VTA (Figures 5A, 6A). hM3Dq/hM4Di was engineered from muscarinic receptors that specifically respond to clozapine N-oxide (CNO, MedChem Express; Armbruster et al., 2007). Three weeks after surgery, CNO (1 mg/kg, i.p.) or saline (0.9%, equal volume, i.p.) was injected 1 h before the behavioral test and EEG recording. Saline was injected in the control group. In the behavioral testing, sevoflurane (2.4%) with oxygen (1 L/min) was used for induction 5 min before LORR, and EEG signals were collected. After LORR, sevoflurane anesthesia (2.4%) was maintained for 30 min, and then, behavioral changes were observed 5 min after RORR (Figures 5C,H,I, 6C,H,I).

### *In vivo* Optogenetic Stimulation During Sevoflurane Anesthesia

**Figure 7:**
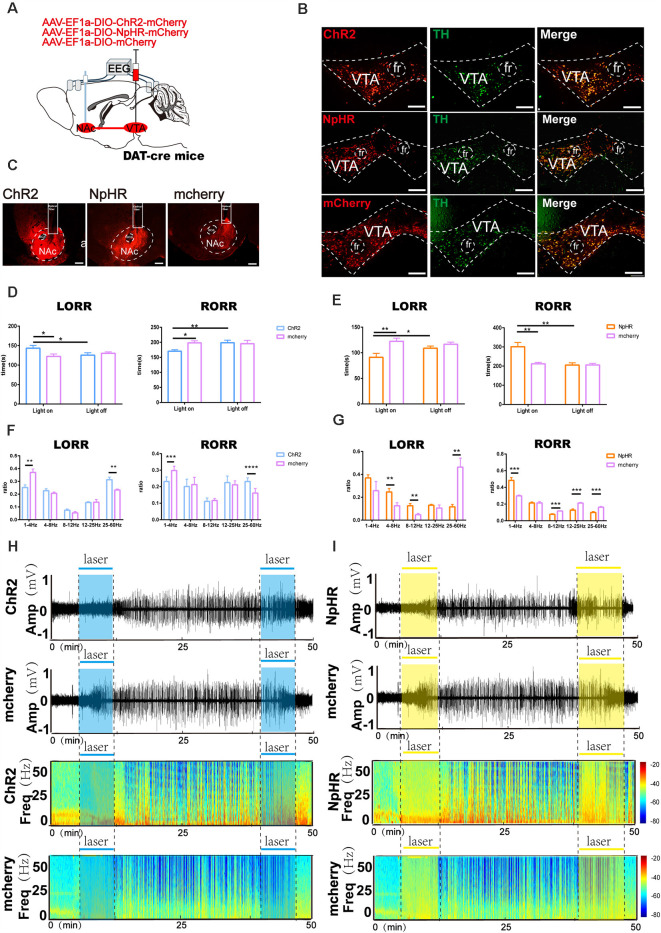
Optogenetic activation or inhibition of VTA^DA^-NAc projections modulate the process of sevoflurane anesthesia. **(A)** Schematic of optogenetic virus injection and stimulation sites. **(B)** Expression of ChR2/NpHR/mCherry (red) and TH immunofluorescence (green) in the VTA. **(C)** Expression of VTA-DA neuron terminals in the NAc. **(D)** Optical stimulation of VTA dopaminergic projections in the NAc prolonged the induction time and reduced the emergence time. **(E)** Optical inhibition of dopaminergic terminals in the NAc reduced the induction time and prolonged the emergence time. **(F)** Optogenetic activation of dopaminergic terminals mediated the band distribution of EEG power during both the LORR and RORR process. **(G)** Optogenetic inhibition of VTA dopaminergic projection to the NAc mediated the band distribution of EEG power during both the LORR and RORR processes. **(H,I)** Representative EEG traces and heat map from each group. Data are presented as the mean ± SD. *p < 0.05, **p < 0.01, ***p < 0.001, and ****p < 0.0001, *n* = 8, by paired and unpaired *t*-test.

We also used optogenetic methods to examine the causal role of the VTA^DA^ during sevoflurane anesthesia. We implanted an optic fiber into the VTA and injected rAAV-EF1a-DIO-ChR2-mCherry into the VTA of DAT-cre mice. To test the vital role of VTA^DA^-NAc projections in sevoflurane anesthesia, we injected rAAV-EF1a-DIO-ChR2-mCherry/rAAV-EF1a-DIO-NpHR-mCherry/rAAV-EF1a-DIO-mCherry into the VTA of DAT-cre mice and implanted an optic fiber into the NAc. An intensity division cube (200 mm/0.37 numerical aperture, Newton Inc., China) was connected to the laser output of a 473 nm optogenetics system (Intelligent Light System, Newdoon Inc., China). The optical power at the tip of the fiber was tested with an optical power meter (PM100D, Thorlabs) and was calibrated to 10–15 mW. For optical stimulation of VTA-DA neurons and their termini, pulses of 473 nm light with a 15 ms width at 20 Hz and pulses of 589 nm light with a 20 ms width at 10 Hz were applied (Taylor et al., 2016) during the LORR and RORR periods, respectively. The optogenetic activation mode was started during the LORR and RORR periods. After 2.4% sevoflurane induced LORR for 5 min, mice were observed for 5 min to determine whether the righting reflex was restored (Figure 2C). We also observed behavioral and EEG changes from optogenetic activation (Figures 7H,I).

### EEG Recording and Spectral Analysis

EEG signals were recorded at least 5 days after the behavioral test to allow for recovery from anesthesia. The multichannel signal acquisition system (Apolo, Bio-Signal Technologies, USA) was used to acquire EEG signals. The EEG signals were collected and filtered between 0.1 and 300 Hz. Before induction, the EEG signals were recorded for 5 min. Then, the EEG signals were continuously recorded from 5 min after administration to recovery from sevoflurane anesthesia, including anesthesia maintained for 30 min. For optogenetic and chemogenetic experiments, power spectrum analysis was conducted on data from the period of induction and the recovery period during sevoflurane anesthesia. The relative powers in the different frequency bands were computed by averaging the signal power across the frequency range of each band (δ: 1 to 4 Hz, θ: 4 to 8 Hz, α: 8 to 12 Hz, β: 12–25 Hz, and γ: 25 to 60 Hz) and then dividing by the total power from 1 to 60 Hz as described in previous studies (Liu et al., 2020; Luo et al., 2020). The spectrogram was bandpass filtered from 1–60 Hz. Spectrograms were constructed using multitaper methods implemented using the Chronux toolbox in MATLAB 2016a (MathWorks, Cambridge, UK).

### Histological Verification

Mice were deeply anesthetized with isoflurane before transcardial infusion of 50 ml PBS followed by 4% PFA. The brains were harvested, postfixed overnight at 4°C, soaked in 30% sucrose in PBS at 4°C until sinking, and then coronally sectioned into 30-μm slices on a cryostat (Leica CM1950). All the brain sections were first incubated in a blocking solution (PBS containing 2.5% normal goat serum, 1.5% bovine serum albumin and 0.1% Triton™ X-100) for 2 h at room temperature and were then incubated with a primary antibody (rabbit c-Fos antibody, 1:200; anti-tyrosine hydroxylase antibody, 1:1,000) in blocking solution overnight at 4°C, followed by a 3 × 10 min wash with PBST (PBS with 0.1% Triton X-100, vol/vol). The sections were then incubated with a secondary antibody (goat anti-rabbit conjugated to Alexa 488/594, 1:1,000 dilution) at room temperature for 2 h. After another 3 × 10 min wash with PBST, the sections were mounted on glass slides and covered with mounting media (Gold antifade reagent with DAPI, Life Technologies, USA). Images of immunostaining were captured on an Olympus BX63 Virtual Microscopy System (Figure 2B).

For the c-Fos experiment, we stained c-Fos in three groups (*n* = 6 for each group). In the anesthesia group, C57BL/6 mice were kept in an anesthesia state in a box with a constant level of sevoflurane (2.4%) and oxygen (1 L/min) for 2 h. For the recovery group, mice were kept awake at room temperature for 2 h after administering sevoflurane (2.4%) and oxygen (1 L/min) for 2 h. For the wakefulness group, we performed c-Fos staining with no operation. The mice were then anesthetized with 1% pentobarbital (50 mg/kg, i.p.) injection. The histological protocol we employed for localization of the cannula position and immunofluorescence was the same as previously mentioned.

### Statistical Analysis

All statistical analyses were performed by GraphPad Prism software package, version 6.0 (GraphPad Software Inc., San Diego, CA, USA). All data were subject to normality tests. One-way analysis of variance (ANOVA) followed by Bonferroni’s test for the three groups of c-Fos staining were used. The differences in the LORR and RORR times and the EEG band percentage between groups were also detected using unpaired Student’s *t*-test. Paired Student’s *t-tests* were used to analyze the differences in calcium signals and DA neurotransmitter signals between pre- and post-events. Data are presented as the mean ± SEM or mean ± SD. In all cases, *p-values* < 0.05 were considered significant.

## Results

### VTA Activity and Neural Dynamics in Response to Sevoflurane Anesthesia

To determine the role of the VTA in sevoflurane-induced general anesthesia, we first observed c-Fos expression as a marker of activated neurons during the state of wakefulness, sevoflurane-induced anesthesia, and recovery from sevoflurane anesthesia. We found that, compared with that induced in sevoflurane anesthesia groups, the increase of the amount of c-Fos expression induced in the wakefulness groups, was less than that induced in the recovery from sevoflurane anesthesia groups (wakefulness group: 225.5 [56.2] vs. sevoflurane-induced anesthesia groups: 30.83 [7.3], *p =* 0.0251, *n* = 6, recovery from sevoflurane anesthesia groups: 319.5 [29.2] vs. sevoflurane-induced anesthesia groups: 30.83 [7.3], *p =* 0.0015, *n* = 6, *F*_(2, 15)_ = 9.995; Figures 1A,B). This result may suggest that neurons in the VTA were inhibited during sevoflurane anesthesia and activated during the recovery process from sevoflurane anesthesia.

Moreover, to investigate the real-time activity of VTA-DA neurons during sevoflurane anesthesia, we injected cre-dependent rAAV-hSyn-DIO-GCaMP6s into the VTA of DAT-cre mice (Figures 1D,E) and used fiber photometry to record changes in the Ca^2+^ signals *in vivo* during isoflurane anesthesia (Figure 1A). During induction of sevoflurane anesthesia, we analyzed the Ca^2+^ signals in three periods: wake period (−300 to −150 s), induction period (−150–0 s), and anesthesia period (0–150 s). The Ca^2+^ signals noticeably decreased after sevoflurane-induced LORR (*p* = 0.0006, *n* = 8) and during sevoflurane anesthesia (*p* = 0.0008, *n* = 8). In the recovery period, three time periods were analyzed, including the anesthesia period (−300 to −150 s), recovery period (−150–0 s), and wake period (0–150 s). A robust increase due to RORR was observed (*p* = 0.025, *n* = 8; Figures 1F–H). These findings indicate that the activity of DA neurons in the VTA is altered under sevoflurane anesthesia.

### Activation of VTA-DA Neurons Modulates the Induction of and Emergence From Sevoflurane Anesthesia

We further determined the role of VTA-DA neurons. Here, we employed optogenetics to manipulate the activity of VTA-DA neurons in DAT-cre mice combined with behavioral tests and EEG recordings (Figure 2A). Compared with the mCherry group, photo stimulation of dopaminergic neurons in the VTA was given at the onset of sevoflurane until LORR, which prolonged the LORR time (ChR2-light-on: 112.7 s vs. mCherry-light-on: 100.8 [9.7] s, *p* = 0.045, *n* = 8). When stimulation was started at the end of sevoflurane inhalation and continued until RORR, optical activation of VTA-DA neurons shortened the arousal time (ChR2-light-on: 168.1 [22.4] s vs. mCherry-light-on: 205.8 [40.2] s, *p* = 0.0047, *n* = 8). Within each group, laser stimulation notably prolonged the LORR time (ChR2-light-on: 112.7 [12.0] s vs. ChR2-light-off: 97.8 [7.7] s, *p* = 0.045, *n* = 8) and shortened the RORR time (ChR2-light-on: 168.1 [22.4] s vs. ChR2-light-off: 208.2 [21.7] s, *p* = 0.0052, *n* = 8), compared with the light-off control (Figure 2D). Moreover, the EEG recordings demonstrated a significant difference in the power spectrum between the ChR2 group and mCherry group during the LORR period, including the ratio of δ bands (ChR2-light-on: 24.7 [5.7] % vs. mCherry-light-on: 31.2 [2.3] %, *p* = 0.008, *n* = 8), β bands (ChR2-light-on: 17.5 [4.5] % vs. mCherry-light-on: 12.9 [2.3] %, *p* = 0.04, *n* = 8), and γ bands (ChR2-light-on: 25.4 [3.5] % vs. mCherry-light-on: 18.2 [3.5] %, *p* = 0.002, *n* = 8). Meanwhile, during emergence to RORR, a strikingly decreased ratio of θ bands (ChR2-light-on: 20.07 [2.6] % vs. mCherry-light-on: 28.2 [5.6] %, *p* = 0.0037, *n* = 8) and increased ratio of β bands (ChR2-light-on: 23.2 [2.9] % vs. mCherry-light-on: 19.6 [1.7] %, *p* = 0.014, *n* = 8) and γ bands (ChR2-light-on: 23.7 [8.1] % vs. mCherry-light-on: 12.8 [9.3] %, *p* = 0.004, *n* = 8) were found between ChR2 group and mCherry group (Figure 2E). It is clear that the bands of high-frequency waves were enhanced and the bands of low-frequency waves were attenuated in the ChR2 group with blue light during both the LORR and RORR processes (Figures 2F,G). Taken together, the results indicate that activation of VTA-DA neurons modulate the processes of induction of and emergence from sevoflurane anesthesia.

### Dopamine in the NAc Is Altered by the State of Sevoflurane Anesthesia

Our above results show that VTA-DA neurons are involved in modulating the state of sevoflurane anesthesia. In our previous study, we found that D1 receptors in the NAc were involved in modulating the process of emergence from general anesthesia (Zhang et al., 2020, 2021). To investigate whether sevoflurane had an influence on the change in the level of dopamine neurotransmitters in VTA-related downstream NAc areas, we measured the dynamics of the extracellular dopamine concentrations during sevoflurane anesthesia in the mouse NAc. Three periods were analyzed: the wake period (−300 to −150 s), induction period (−150–0 s), and early anesthesia period (0–150 s). During the period from sevoflurane induction to LORR, statistical comparison indicated that the DA transmitters amount in the NAc in the anesthesia period were dramatically lower than the wake period (*p* = 0.000085, *n* = 10).

During emergence from sevoflurane anesthesia, we analyzed three time periods: the anesthesia period (−300 to −150 s), recovery period (−150–0 s), and wake period (0–150 s). A robust increase in the NAc DA transmitters’ fluorescence signal was observed during the process of RORR. The DA transmitters in the NAc in the recovery period showed a significant increase compared to anesthesia period (*p* = 0.0014, *n* = 10; Figures 3D–F). These findings indicate that the release of DA transmitters in the NAc is also regulated by sevoflurane anesthesia with the same trend as the activity of VTA-DA neurons.

### NAc Activity and Neural Dynamics in Response to Sevoflurane Anesthesia

We used a fiber photometry recording of Ca^2+^ activity and the expression of c-Fos in NAc neurons to examine the correlation between NAc neural activity and sevoflurane anesthesia. We observed c-Fos expression during the state of wakefulness, sevoflurane-induced anesthesia, and recovery from sevoflurane anesthesia. Compared with the sevoflurane anesthesia groups, statistical analysis of the amount of c-Fos expression demonstrated a significant increase in the wakefulness groups and the groups that recovered from sevoflurane anesthesia (wakefulness groups: 84.8 [18.1] vs. sevoflurane-induced anesthesia groups: 14.1 [3.5], *p* = 0.0416, *n* = 8; recovery from sevoflurane anesthesia groups: 140.3 [22.1] vs. sevoflurane anesthesia-induced groups: 14.1 [3.5], *p* = 0.0006, *n* = 8, *F*_(2,15)_ = 11.63; Figures 4A,B). This result might suggest that neuronal activity in the NAc could be altered by the stages of sevoflurane anesthesia. Moreover, to record the specific and temporary change in neuronal activity of NAc neurons, we injected rAAV-hSyn-GCaMP6s into the C57BL/6 mice (Figure 4D) and used fiber photometry to record changes in the Ca^2+^ signals *in vivo* during sevoflurane anesthesia. Then we analyzed calcium signals in three periods: the wake period (−300 to −150 s), induction period (−150–0 s), and early anesthesia period (0–150 s). The Ca^2+^ signal decreased with the sevoflurane-induced LORR process (*p* = 0.01, *n* = 8) and during sevoflurane anesthesia (*p* = 0.005, *n* = 8). In the emergence process, three periods, the anesthesia period (−300 to −150 s), recovery period (−150–0 s), and wake period (0–150 s), were analyzed. A striking increase was observed in the wake stage (*p* = 0.029, *n* = 8; Figures 4E–G). These findings indicate that the activity of neurons in the NAc is altered under sevoflurane anesthesia.

### Activation of the VTA-NAc Pathway Alters the Induction and Emergence Stages From Sevoflurane Anesthesia

The change in Ca^2+^ activity measured in VTA and NAc neurons and the extracellular DA transmitters had a consistent trend of changes during sevoflurane anesthesia, suggesting that the VTA^DA^-NAc pathway might induce a neural activity–dependent release of DA to regulate sevoflurane anesthesia. To selectively activate the VTA-NAc pathway, we injected rAAV-Ef1α-DIO-hM3Dq-EGFP or rAAV-Ef1α-DIO-EGFP vectors into the VTA and performed rAAV/retro-hSyn-cre-mCherry-WPRE-hGH retrograde tracing in the NAc, as previously described (Ren et al., 2018; Coffey et al., 2020). The results showed that retrograde tracing of the virus from NAc-labeled VTA neurons, 72.3% ± 5.9% (SEM) expressed tyrosine hydroxylase by hM3Dq-positive neurons, which implied that the NAc mainly innervated dopaminergic neurons in the VTA (Figure 5B). Compared with the CNO-EGFP or saline-hM3Dq groups, CNO-mediated hM3Dq activation of VTA^DA^-NAc pathways significantly prolonged the LORR tim e (CNO-hM3Dq: 144.4 [17.5] s vs. CNO-EGFP: 115.0 [10.8] s, *p* = 0.0021, CNO-hM3Dq: 144.4 [17.5] s vs. saline-hM3Dq: 117.3 [12.6] s, *p* = 0.0049, *n* = 8) and shortened the RORR time (CNO-hM3Dq: 179.9 [13.1] s vs. CNO-EGFP: 218.5 [15.6] s, *p* = 0.0002, *n* = 8, CNO-hM3Dq: 179.9 [13.1] s vs. saline-hM3Dq: 211.0 [22.3] s, *p* = 0.0067, *n* = 8; Figures 5D,F).

Moreover, EEG analysis showed a similar trend. CNO-mediated hM3Dq activation of VTA^DA^-NAc neurons decreased δ bands (CNO-hM3Dq: 24.7 [2.8] % vs. CNO-EGFP: 32.1 [3.1] %, *p* = 0.0004, *n* = 8) and increased γ bands (24.8 [10.3] % vs. CNO-EGFP: 23.2 [13.2] %, *p* = 0.0119, *n* = 8) during LORR, while it decreased δ bands (CNO-hM3Dq: 25.4 [2.2] % vs. CNO-EGFP:

31.6 [1.0] %, *p* = 0.0003, *n* = 8) and increased γ bands (CNO-hM3Dq: 19.2 [4.0] % vs. CNO-EGFP: 13.5 [2.5] %, *p* = 0.0342, *n* = 8) during RORR (Figures 5F,G).

### Inhibition of the VTA-NAc Pathway Regulates the Induction and Emergence Process of Sevoflurane Anesthesia

After chemogenetic virus injection, 75.8% ± 8.7% (SEM) of neurons expressing Th positive neurons were infected with hM4Di virus (Figure 6B). Compared with the CNO-EGFP group, CNO-mediated hM4Di inhibition of VTA-NAc neurons significantly reduced the LORR time (CNO-hM4Di: 102.4 [11.3] s vs. CNO-EGFP: 121.5 [9.4] s; *p* = 0.0039, *n* = 8) and prolonged the RORR time (CNO-hM4Di: 398.8 [51.5] s vs. CNO-EGFP: 165.4 [35.0] s; *p* = 0.015, *n* = 8, Figures 6D,E).

In addition, EEG analysis showed that compared to CNO-EGFP, CNO-mediated hM4Di inhibition of VTA-NAc neurons significantly increased the ratio of the δ band (CNO-hM4Di: 53.7 [8.2] % vs. CNO-EGFP: 35.5 [9.9] %, *p* = 0.008, *n* = 8) but reduced the β band (CNO-hM4Di: 8.9 [2.6] % vs. CNO-EGFP: 12.1 [3.0] %, *p* = 0.049, *n* = 8) and γ band (8.0 [3.6] % vs. CNO-EGFP: 18.4 [8.2] %, *p* = 0.0056, *n* = 8) during LORR (Figure 6F). During the RORR time, CNO led to an increased the ratio of the δ band (CNO-hM4Di: 50.2 [1.1] % vs. CNO-EGFP: 31.1 [1.8] %, *p* = 0.0006, *n* = 8) and decreased the α band (CNO-hM4Di: 8.8 [2.4] % vs. CNO-EGFP: 12.12 [0.8] %, *p* = 0.0029, *n* = 8), β band (CNO-hM4Di: 19.6 [4.3] % vs. CNO-EGFP: 11.8 [3.7] %; *p* = 0.0002, *n* = 8) and γ band (CNO-hM4Di: 7.8 [3.2] % vs. CNO-EGFP: 15.5 [0.1] %, *p* = 0.00004, *n* = 8), as shown in Figures 6E–G.

These results may reveal that VTA-NAc projections play a crucial role in the modulation of the induction and emergence processes of sevoflurane anesthesia.

### Optical Stimulation and Inhibition of Dopaminergic Terminals of VTA-DA Neurons in the NAc Modulate the Process of Sevoflurane Anesthesia

To test the participation of VTA dopaminergic fiber projections to the NAc in sevoflurane anesthesia, we implanted a fiber optic probe above the NAc of DAT-cre mice following AAV-EF1a-DIO-ChR2-mCherry, AAV-EF1a-DIO-NpHR-mCherry or AAV-EF1a-DIO-mCherry virus infusion into the VTA (Figures 7A–C).

Compared with the controls that did not receive laser stimulation, photo stimulation of dopaminergic terminals in the NAc after sevoflurane inhalation prolonged the LORR time (ChR2-light-on: 145.5 [14.3] s vs. mCherry-light-on: 120.6 [10.2] s, *p* = 0.0047, *n* = 8) and reduced the RORR time (ChR2-light-on: 168.3 [12.3] s vs. mCherry-light-on: 197.3 [19.5] s, *p* = 0.0131, *n* = 8). Additionally, compared with the mCherry group, photostimulation prolonged the LORR time (ChR2-light-on: 145.5 [14.3] s vs. mCherry-light-on: 120.6 [10.2] s, *p* = 0.0433, *n* = 8) and reduced the RORR time (ChR2-light-on: 168.3 [12.3] s vs. mCherry-light-on: 197.3 [19.5] s, *p* = 0.0045, *n* = 8) in the ChR2 group, as shown in Figure 7D. Conversely, compared with mCherry, photoinhibition of VTA dopaminergic projections in the NAc reduced the LORR time (NpHR -light-on: 98.5 [20.4] s vs. mCherry-light-on: 122.4 [16.3] s, *p* = 0.007, *n* = 8) and prolonged the RORR time in the NpHR group(NpHR-light-on: 303.3 [60.0] s vs. mCherry-light-on: 212.3 [21.1] s, *p* = 0.002, *n* = 8; Figure 7E).

During the LORR process, EEG analysis demonstrated that optical activation significantly decreased the δ band percentage (25.6 [6.4] % vs. 36.7 [6.8] %, *p* = 0.0035, *n* = 8) and increased the power of the γ band (32.2 [4.3] % vs. 27.7 [6.2] %, *p* = 0.0021, *n* = 8) between the mCherry group and the ChR2 group. During the RORR process, photostimulation of the VTA dopaminergic projections in the NAc significantly decreased the power of the δ band (23.2 [2.5] % vs. 29.8 [2.4] %, *p* = 0.0002, *n* = 8) and increased the γ band (22.9 [2.0] % vs. 16.1 [3.3] %, *p* = 0.00006, *n* = 8) in the ChR2 group (Figure 7F). Nevertheless, photoinhibition increased the power of the δ band (36.7 [7.0] % vs. 29.7 [2.6] %, *p* = 0.0311, *n* = 8) and decreased the β band (13.0 [2.3] % vs. 21.2 [4.3] %, *p* = 0.000009, *n* = 8; Figure 7G). These behavioral and EEG findings indicate that the induction and emergence processes of sevoflurane anesthesia are modulated by VTA^DA^-NAc projections.

## Discussion

In this study, we manipulated VTA-DA neurons to elucidate their regulatory role in sevoflurane anesthesia using calcium fiber photometry recordings and optogenetics. The results revealed that VTA-DA neurons were involved in sevoflurane anesthesia, and targeted activation of VTA-DA neurons resulted in a longer induction time and shorter emergence time associated with sevoflurane anesthesia. The neural activity and extracellular dopamine levels of the NAc were correlated with sevoflurane-anesthetized states, and NAc signal fluctuations might be caused by changes in VTA-releasing dopamine neurotransmitters. Moreover, using chemogenetic and optogenetic approaches, activation of VTA dopaminergic projections to the NAc facilitated arousal according to both behavioral and simultaneous EEG signal changes under sevoflurane anesthesia. Our results demonstrate that VTA-DA neurons play a critical role in modulating sevoflurane anesthesia *via* the VTA^DA^-NAc pathway.

We found that the neuronal activity of VTA-DA decreased under sevoflurane anesthesia and increased during recovery from sevoflurane anesthesia by detecting the expression of the c-Fos protein and using calcium fiber photometry recordings, which indicated that sevoflurane anesthesia may require an inhibition of VTA neurons to some degree. In the recovery period, a positive trend was observed in the transition from anesthesia to wakefulness, suggesting that VTA neurons were highly activated at the moment of emergence. Similar results were observed in a previous study, in which VTA neurons exhibited increasing firing rates during wakefulness, and the same population of neurons displayed reduced firing rates during anesthesia and slow-wave sleep (Yanagihara et al., 2020). Moreover, using fiber photometry recordings to activate VTA-DA neurons led to a decline before the wakefulness to nonrapid eye movement (NREM) sleep transition, and the activity of VTA-DA neurons augmented their activity before NREM-to-arousal transitions (Eban-Rothschild et al., 2016). We found that optogenetic activation of VTA-DA neurons by optical stimulation promoted the arousal of anesthesia and increased the powers of the β and γ bands in ChR2+ mice during sevoflurane anesthesia. Similar evidence was provided that optical VTA stimulation during isoflurane anesthesia produced behavioral and EEG evidence of arousal and restored the righting reflex and that pretreatment with the DA receptor antagonist before optical VTA stimulation inhibited the arousal responses and restoration of righting in all mice (Taylor et al., 2016). Selective bilateral VTA-DA neuron lesions significantly prolong the recovery time from propofol in rats (Zhou et al., 2015). VTA non-DA neurons show increased firing rates during active wakefulness and rapid eye movement sleep relative to quiet wakefulness. Adequate anesthesia produces a significantly reduced VTA GABAergic (VTA-GABA) neuron firing rate (Lee et al., 2001). Owing to its role in sleep–wake regulation, as well as in general anesthesia of VTA-DA neurons, the VTA is well-positioned for modulating general anesthesia.

How do dopaminergic neurons in the VTA have a waking-promoting effect? As one of the major targets of the VTA-NAc, the VTA has been thoroughly studied for its regulation of the sleep-wake cycle and general anesthesia. Many studies have shown that a subpopulation of DA neurons in the VTA project to the NAc (Beier et al., 2015; Qi et al., 2016; Breton et al., 2019; Mingote et al., 2019; Tu et al., 2020). Recent behavioral research has shown that DA terminals in the ventral NAc medial shell are excited by unexpected aversive outcomes and to cues that predict them, whereas DA terminals in other NAc subregions are persistently depressed (de Jong et al., 2019). Another study showed that activating the VTA dopaminergic circuit could mimic tonic DA release in the NAc, which could inhibit reward consummatory behavior (Mikhailova et al., 2016). Eban-Rothschild et al. (2016) found that selective photostimulation of VTA projections to the NAc, the central amygdala and the dorsal-lateral striatum during NREM sleep induced a significant reduction in the latency to wake. However, the VTA-NAc pathway was the only projection that could induce mice to maintain wakefulness during the first 6 h of the light phase, suggesting that the VTA-NAc pathway plays a critical role in arousal (Eban-Rothschild et al., 2016). Recently, Luo et al. (2018) showed that optogenetic activation of NAc D1 receptorneurons (NAc-D1R) induces an immediate transition from non-rapid eye movement sleep to wakefulness and that chemogenetic stimulation prolongs arousal (Luo et al., 2018). The NAc participates in maintaining consciousness, and activation of the NAc potentiates the response to a general anesthetic (Ma and Leung, 2006). A previous study found that NAc-D1R in the NAc was involved in regulating the process of emergence from general anesthesia in aged mice (Zhang et al., 2020, 2021). These findings suggest that the NAc apparently regulates general anesthesia. However, few studies have explored the relationship between VTA^DA^-NAc projections and sevoflurane anesthesia in great depth. Therefore, we proposed that VTA-induced arousal under anesthesia is mediated by the VTA-NAc pathway.

We next manipulated the VTA^DA^-NAc pathways to examine their regulatory effects on sevoflurane anesthesia by chemogenetic and optogenetics technology. Our results show that activation of the VTA^DA^-NAc pathways modulated sevoflurane induction and emergence and decreased the depth of anesthesia during the LORR and RORR periods. The current results uncover a fundamental role for the VTA dopaminergic circuitry in the maintenance of the awake state and ethologically relevant sleep-related behaviors (Eban-Rothschild et al., 2016; Oishi and Lazarus, 2017; Yin et al., 2019). Furthermore, increased activation of the VTA-DA pathway relieves the inhibitory effect of cortices within GABA neurons by resisting projections from the NAc GABAergic to cortical regions (Eban-Rothschild et al., 2016; Oishi and Xu, 2017). It also verified the influence of EEG changes in the cortex of anesthetics acting on alterations in VTA^DA^-NAc pathway activity. Therefore, we inferred that the VTA^DA^-NAc pathways are largely responsible for the awakening process of general anesthesia.

We used c-Fos and fiber photometry recordings to show that sevoflurane anesthesia altered NAc neuron activity by suppression during the induction process of sevoflurane anesthesia and activation during the recovery process of sevoflurane anesthesia. Using DA sensors, we detected the DA transmitters in the NAc under sevoflurane anesthesia and demonstrated that the level of the DA transmitters declined with sevoflurane anesthesia induced to LORR, remained constant during the anesthesia period, and increased during the anesthesia-to-wake transition. Another study indicated that propofol increased the concentration of DA in the NAc using *in vivo* brain microdialysis (Pain et al., 2002). The elevated levels of DA transmitters during waking and REM sleeping the NAc could result from changes during these two states in afferent modulation at the level of cell bodies or at the level of dopaminergic terminals (Léna et al., 2005), suggesting that afferents of upstream dopaminergic nuclei participate in the process of sevoflurane anesthesia. Our study provides evidence of the direct regulatory effect of dopamine on NAc neurons during anesthesia emergence. In summary, we provide multiple lines of evidence to support the idea that VTA-DA has a crucial influence on modulating sevoflurane anesthesia *via* the VTA-NAc dopaminergic pathways.

The present study has some limitations. First, the NAc includes various GABAergic neurons and other types of neurons, and we were not able to distinguish between the specific receptor neurons in response to DA in the VTA. This question requires the use of selective techniques for further analysis of the function of specific neuron populations. Second, the effects of sevoflurane on global oxidative metabolism and cerebral blood flow (Slupe and Kirsch, 2018) that accompany the induction and recovery periods may also affect the behavioral and EEG results. Finally, the VTA also sends brain-wide projections, including direct projections to the cortex and LH, and the NAc sends projections to the ventral pallidum and the cortex. Some of these pathways have been shown to be involved in the regulation of sleep-wake but have been rarely studied in relation to general anesthesia.

## Data Availability Statement

The raw data supporting the conclusions of this article will be made available by the authors, without undue reservation.

## Ethics Statement

The animal study was reviewed and approved by Nursing Committee of Zunyi Medical University.

## Author Contributions

This work was primarily conceived by YZ and HG. Data were collected by HG and CL and analyzed by HH, CL, and HG. Manuscript was written by HG, CL, HH, JZ, and HC. Figures were produced by HG. All authors contributed to the article and approved the submitted version.

## Conflict of Interest

The authors declare that the research was conducted in the absence of any commercial or financial relationships that could be construed as a potential conflict of interest.

## References

[B1] Armbruster B. N. Li X. Pausch M. H. Herlitze S. Roth B. L. (2007). Evolving the lock to fit the key to create a family of G protein-coupled receptors potently activated by an inert ligand. Proc. Natl. Acad. Sci. U S A 104, 5163–5168. 10.1073/pnas.0700293104 17360345 PMC1829280

[B2] Beier K. T. Steinberg E. E. DeLoach K. E. Xie S. Miyamichi K. Schwarz L.. (2015). Circuit architecture of VTA dopamine neurons revealed by systematic input-output mapping. Cell 162, 622–634. 10.1016/j.cell.2015.07.015 26232228 PMC4522312

[B3] Boonmak P. Boonmak S. Pattanittum P. (2016). High initial concentration versus low initial concentration sevoflurane for inhalational induction of anesthesia. Cochrane Database Syst. Rev. 6:Cd006837. 10.1002/14651858.CD006837.pub3 27356171 PMC8676071

[B4] Breton J. M. Charbit A. R. Snyder B. J. Fong P. T. K. Dias E. V. Himmels P.. (2019). Relative contributions and mapping of ventral tegmental area dopamine and GABA neurons by projection target in the rat. J. Comp. Neurol. 527, 916–941. 10.1002/cne.24572 30393861 PMC6347508

[B5] Brioni J. D. Varughese S. Ahmed R. Bein B. (2017). A clinical review of inhalation anesthesia with sevoflurane: from early research to emerging topics. J. Anesth. 31, 764–778. 10.1007/s00540-017-2375-6 28585095 PMC5640726

[B6] Coffey K. R. Marx R. G. Vo E. K. Nair S. G. Neumaier J. F. (2020). Chemogenetic inhibition of lateral habenula projections to the dorsal raphe nucleus reduces passive coping and perseverative reward seeking in rats. Neuropsychopharmacology 45, 1115–1124. 10.1038/s41386-020-0616-0 31958800 PMC7235029

[B7] de Jong J. W. Afjei S. A. Pollak Dorocic I. Peck J. R. Liu C. Kim C. K.. (2019). A neural circuit mechanism for encoding aversive stimuli in the mesolimbic dopamine system. Neuron 101, 133.e7–151.e7. 10.1016/j.neuron.2018.11.005 30503173 PMC6317997

[B8] Eban-Rothschild A. Rothschild G. Giardino W. J. Jones J. R. de Lecea L. (2016). VTA dopaminergic neurons regulate ethologically relevant sleep-wake behaviors. Nat. Neurosci. 19, 1356–1366. 10.1038/nn.4377 27595385 PMC5519826

[B9] Franks N. P. (2008). General anesthesia: from molecular targets to neuronal pathways of sleep and arousal. Nat. Rev. Neurosci. 9, 370–386. 10.1038/nrn2372 18425091

[B10] Franks N. P. Zecharia A. Y. (2011). Sleep and general anesthesia. Can. J. Anaesth. 58, 139–148. 10.1007/s12630-010-9420-3 21170623

[B11] Harrington M. (2014). Dopamine pathway induces emergence from anesthesia. Lab. Anim. 43:304. 10.1038/laban.619 25141054

[B12] Lee R. S. Steffensen S. C. Henriksen S. J. (2001). Discharge profiles of ventral tegmental area GABA neurons during movement, anesthesia and the sleep-wake cycle. J. Neurosci. 21, 1757–1766. 10.1523/JNEUROSCI.21-05-01757.2001 11222665 PMC6762953

[B14] Léna I. Parrot S. Deschaux O. Muffat-Joly S. Sauvinet V. Renaud B.. (2005). Variations in extracellular levels of dopamine, noradrenaline, glutamate and aspartate across the sleep–wake cycle in the medial prefrontal cortex and nucleus accumbens of freely moving rats. J. Neurosci. Res. 81, 891–899. 10.1093/eurjpc/zwaa114 16041801

[B15] Leung L. S. Luo T. Ma J. Herrick I. (2014). Brain areas that influence general anesthesia. Prog. Neurobiol. 122, 24–44. 10.1016/j.pneurobio.2014.08.001 25172271

[B16] Li J. Li H. Wang D. Guo Y. Zhang X. Ran M.. (2019). Orexin activated emergence from isoflurane anesthesia involves excitation of ventral tegmental area dopaminergic neurones in rats. Br. J. Anaesth. 123, 497–505. 10.1016/j.bja.2019.07.005 31399212

[B17] Liu C. Liu J. Zhou L. He H. Zhang Y. Cai S.. (2021). Lateral habenula glutamatergic neurons modulate isoflurane anesthesia in mice. Front. Mol. Neurosci. 14:628996. 10.3389/fnmol.2021.628996 33746711 PMC7969819

[B18] Liu C. Zhou X. Zhu Q. Fu B. Cao S. Zhang Y.. (2020). Dopamine neurons in the ventral periaqueductal gray modulate isoflurane anesthesia in rats. CNS Neurosci. Ther. 26, 1121–1133. 10.1111/cns.13447 32881314 PMC7564192

[B19] Luo T. Y. Cai S. Qin Z. X. Yang S. C. Shu Y. Liu C. X.. (2020). Basal forebrain cholinergic activity modulates isoflurane and propofol anesthesia. Front. Neurosci. 14:559077. 10.3389/fnins.2020.559077 33192246 PMC7652994

[B21] Luo Y. J. Li Y. D. Wang L. Yang S. R. Yuan X. S. Wang J.. (2018). Nucleus accumbens controls wakefulness by a subpopulation of neurons expressing dopamine D(1) receptors. Nat. Commun. 9:1576. 10.1038/s41467-018-03889-3 29679009 PMC5910424

[B22] Ma J. Leung L. S. (2006). Limbic system participates in mediating the effects of general anesthetics. Neuropsychopharmacology 31, 1177–1192. 10.1038/sj.npp.1300909 16205783

[B23] Mikhailova M. A. Bass C. E. Grinevich V. P. Chappell A. M. Deal A. L. Bonin K. D.. (2016). Optogenetically-induced tonic dopamine release from VTA-nucleus accumbens projections inhibits reward consummatory behaviors. Neuroscience 333, 54–64. 10.1016/j.neuroscience.2016.07.006 27421228 PMC4992643

[B24] Mingote S. Amsellem A. Kempf A. Rayport S. Chuhma N. (2019). Dopamine-glutamate neuron projections to the nucleus accumbens medial shell and behavioral switching. Neurochem. Int. 129:104482. 10.1016/j.neuint.2019.104482 31170424 PMC6855309

[B25] Nelson L. E. Guo T. Z. Lu J. Saper C. B. Franks N. P. Maze M. (2002). The sedative component of anesthesia is mediated by GABA(A) receptors in an endogenous sleep pathway. Nat. Neurosci. 5, 979–984. 10.1038/nn913 12195434

[B26] Nestler E. J. Carlezon W. A. Jr. (2006). The mesolimbic dopamine reward circuit in depression. Biol. Psychiatry 59, 1151–1159. 10.1016/j.biopsych.2005.09.018 16566899

[B27] Oishi Y. Lazarus M. (2017). The control of sleep and wakefulness by mesolimbic dopamine systems. Neurosci. Res. 118, 66–73. 10.1016/j.neures.2017.04.008 28434991

[B28] Oishi Y. Suzuki Y. Takahashi K. Yonezawa T. Kanda T. Takata Y.. (2017). Activation of ventral tegmental area dopamine neurons produces wakefulness through dopamine D2-like receptors in mice. Brain Struct. Funct. 222, 2907–2915. 10.1007/s00429-017-1365-7 28124114

[B29] Oishi Y. Xu Q. (2017). Slow-wave sleep is controlled by a subset of nucleus accumbens core neurons in mice. Nat. Commun. 8:734. 10.1038/s41467-017-00781-4 28963505 PMC5622037

[B30] Pain L. Gobaille S. Schleef C. Aunis D. Oberling P. (2002). in vivo dopamine measurements in the nucleus accumbens after nonanesthetic and anesthetic doses of propofol in rats. Anesth. Analg. 95, 915–919. 10.1097/00000539-200210000-00022 12351267

[B31] Qi J. Zhang S. Wang H. L. Barker D. J. Miranda-Barrientos J. Morales M. (2016). VTA glutamatergic inputs to nucleus accumbens drive aversion by acting on GABAergic interneurons. Nat. Neurosci. 19, 725–733. 10.1038/nn.4281 27019014 PMC4846550

[B32] Qiu M. H. Zhong Z. G. Chen M. C. Lu J. (2019). Nigrostriatal and mesolimbic control of sleep-wake behavior in rat. Brain Struct. Funct. 224, 2525–2535. 10.1007/s00429-019-01921-w 31324969 PMC6713786

[B33] Ren S. Wang Y. Yue F. Cheng X. Dang R. Qiao Q.. (2018). The paraventricular thalamus is a critical thalamic area for wakefulness. Science 362, 429–434. 10.1126/science.aat2512 30361367

[B34] Shen M. Jiang C. Liu P. Wang F. Ma L. (2016). Mesolimbic leptin signaling negatively regulates cocaine-conditioned reward. Transl. Psychiatry 6:e972. 10.1038/tp.2016.223 27922639 PMC5315559

[B35] Skibicka K. P. Shirazi R. H. Rabasa-Papio C. Alvarez-Crespo M. Neuber C. Vogel H.. (2013). Divergent circuitry underlying food reward and intake effects of ghrelin: dopaminergic VTA-accumbens projection mediates ghrelin’s effect on food reward but not food intake. Neuropharmacology 73, 274–283. 10.1016/j.neuropharm.2013.06.004 23770258

[B36] Slupe A. M. Kirsch J. R. (2018). Effects of anesthesia on cerebral blood flow, metabolism and neuroprotection. J. Cereb. Blood Flow Metab. 38, 2192–2208. 10.1177/0271678X18789273 30009645 PMC6282215

[B37] Solt K. Van Dort C. J. Chemali J. J. Taylor N. E. Kenny J. D. Brown E. N. (2014). Electrical stimulation of the ventral tegmental area induces reanimation from general anesthesia. Anesthesiology 121, 311–319. 10.1097/ALN.0000000000000117 24398816 PMC4112744

[B38] Sun F. Zeng J. Jing M. Zhou J. Feng J. Owen S. F.. (2018). A genetically encoded fluorescent sensor enables rapid and specific detection of dopamine in flies, fish and mice. Cell 174, 481.e19–496.e19. 10.1016/j.cell.2018.06.042 30007419 PMC6092020

[B39] Taylor N. E. Van Dort C. J. Kenny J. D. Pei J. Guidera J. A. Vlasov K. Y.. (2016). Optogenetic activation of dopamine neurons in the ventral tegmental area induces reanimation from general anesthesia. Proc. Natl. Acad. Sci. U S A 113, 12826–12831. 10.1073/pnas.1614340113 27791160 PMC5111696

[B40] Tu Y. Bi Y. Zhang L. Wei H. Hu L. (2020). Mesocorticolimbic pathways encode cue-based expectancy effects on pain. J. Neurosci. 40, 382–394. 10.1523/JNEUROSCI.1082-19.2019 31694965 PMC6948945

[B41] van der Meij J. Martinez-Gonzalez D. Beckers G. J. L. Rattenborg N. C. (2019). Neurophysiology of avian sleep: comparing natural sleep and isoflurane anesthesia. Front. Neurosci. 13:262. 10.3389/fnins.2019.00262 30983954 PMC6447711

[B42] Xu W. Wang L. Yuan X. S. Wang T. X. Li W. X. Qu W. M.. (2020). Sevoflurane depresses neurons in the medial parabrachial nucleus by potentiating postsynaptic GABA(A) receptors and background potassium channels. Neuropharmacology 181:108249. 10.1016/j.neuropharm.2020.108249 32931816

[B43] Yanagihara S. Ikebuchi M. Mori C. Tachibana R. O. Okanoya K. (2020). Arousal state-dependent alterations in neural activity in the zebra finch VTA/SNc. Front. Neurosci. 14:897. 10.3389/fnins.2020.00897 32973441 PMC7472990

[B44] Yin L. Li L. Deng J. Wang D. Guo Y. Zhang X.. (2019). Optogenetic/chemogenetic activation of GABAergic neurons in the ventral tegmental area facilitates general anesthesia *via* projections to the lateral hypothalamus in mice. Front. Neural Circuits 13:73. 10.3389/fncir.2019.00073 31798420 PMC6878851

[B45] Zhang Y. Gui H. Duan Z. Yu T. Zhang J. Liang X.. (2021). Dopamine D1 receptor in the nucleus accumbens modulates the emergence from propofol anesthesia in rat. Neurochem. Res. [Online ahead of print]. 10.1007/s11064-021-03284-3 33683630

[B46] Zhang Y. Gui H. Hu L. Li C. Zhang J. Liang X. (2020). Dopamine D1 receptor in the NAc shell is involved in delayed emergence from isoflurane anesthesia in aged mice. Brain Behav. 11:e01913. 10.1002/brb3.1913 33094567 PMC7821614

[B47] Zhou X. Wang Y. Zhang C. Wang M. Zhang M. Yu L.. (2015). The role of dopaminergic VTA neurons in general anesthesia. PloS One 10:e0138187. 10.1371/journal.pone.0138187 26398236 PMC4580504

